# Assessment indicators of ovarian response during controlled ovarian stimulation: influencing factors and clinical value

**DOI:** 10.3389/fendo.2026.1793202

**Published:** 2026-05-07

**Authors:** Huishu Xu, Hua Feng, Xiaofeng Liu, Hongmei Yin, Xiuju Wang, Zhaoyue Luo, Yixuan Ma, Guowei Cai, Mengyuan Li, Yanlin Wang, Kexin Lu, Yang Fan, Lei Han

**Affiliations:** 1 Department of Reproductive Medicine, Binzhou Medical University Hospital, Binzhou Medical University, Binzhou, Shandong, China; 2 Department of Clinical Nutrition, Chongqing University Jiangjin Hospital, Chongqing, China; 3 National Drug Clinical Trial Institution of Binzhou Medical University Hospital, Binzhou Medical University, Binzhou, Shandong, China; 4 Department of Gynecology and Obstetrics, The First Affiliated Hospital (Southwest Hospital) of Army Medical University, Chongqing, China; 5 Department of Obstetrics and Gynecology, Binzhou Medical University Hospital, Binzhou, Shandong, China

**Keywords:** controlled ovarian stimulation, follicle output rate, Follicle-To-Oocyte index, integrated prediction framework, ovarian response, ovarian response prediction index, ovarian sensitivity index

## Abstract

Ovarian response to controlled ovarian stimulation (COS) is a core determinant of assisted reproductive technology (ART) success, with marked interindividual variability. Accurate and individualized prediction is essential to optimize treatment safety and efficacy. Traditional static predictors include age, BMI, AMH, AFC, FSH/LH ratio, and inhibin B. Recently, dynamic indices such as FORT, FOI, OSI, and ORPI have improved real time evaluation of ovarian responsiveness. Genetic polymorphisms, epigenetic regulation, and environmental exposures further modulate ovarian sensitivity to gonadotropins. This review proposes a novel Integrated Multi-dimensional Ovarian Response Prediction (IMORP) framework that combines static reserve, dynamic responsiveness, genetics, and environment to guide personalized COS. We grade evidence levels, critically appraise strengths and limitations of each marker, and provide a head to head comparison of dynamic indices. Future directions should integrate multi omics data and clinical parameters to develop molecularly driven predictive models, advancing ART from empirical practice to precision medicine.

## 1 Introduction

According to the 2023 definition established by the American Society for Reproductive Medicine (ASRM), infertility is a disease characterized by the failure to achieve pregnancy and/or the need for medical intervention to attain a successful pregnancy. For patients who fail to conceive after regular, unprotected intercourse, evaluation should be initiated at 12 months if the female partner is under 35 years of age, and at 6 months if she is aged 35 or older (1). Infertility affects reproductive-aged couples globally across all regions. Timely treatment can enhance pregnancy rates, improve psychosocial well-being, and prevent potential reproductive disorders, thereby holding significant implications for both individual and public health. As a critical therapeutic approach for infertility, assisted reproductive technology—primarily represented by *in vitro* fertilization and embryo transfer (IVF-ET)—has achieved remarkable advancements, enabling numerous infertile couples to achieve parenthood.

Among these procedures, ovarian stimulation constitutes a critical component of *in vitro* fertilization technology. It aims to induce multi-follicular development through the administration of exogenous Gn, thereby generating a sufficient number of oocytes. An optimal ovarian response to COS is a prerequisite for successful pregnancy, as it increases opportunities to retrieve more oocytes and perform multiple embryo transfers within a single stimulation cycle, ultimately enhancing cumulative live birth rates (2, 3). Suboptimal ovarian response may lead to compromised oocyte quality or reduced yield, consequently increasing cycle cancellation rates and decreasing pregnancy rates. Conversely, excessive ovarian stimulation elevates serum estrogen levels, potentially triggering life-threatening complications such as Ovarian Hyperstimulation Syndrome (OHSS). This also impairs endometrial receptivity, ultimately resulting in higher cancellation rates for fresh embryo transfers (4).

Determinants of ovarian response encompass multiple clinical parameters: maternal age, body mass index (BMI), ovarian reserve, baseline hormone levels, duration and etiology of infertility, pretreatment regimens prior to COS, selection of COS protocols (e.g., antagonist, long agonist, or progestin-primed ovarian stimulation, Gn types, initial Gn starting doses, and Gn dosage adjustments during treatment. Within ART, established pretreatment strategies include (1): Combined Oral Contraceptives (COC), which synchronize follicular development by suppressing the hypothalamic-pituitary-ovarian axis (HPOA) to reduce endogenous FSH and LH secretion (2); Gonadotropin-releasing hormone(GnRH) agonists that prevent premature LH surges through pituitary desensitization (3); Androgen and growth hormone(GH) supplementation—where androgens enhance follicular recruitment by upregulating FSH receptor expression, while GH improves oocyte mitochondrial function to elevate embryo quality, specifically targeting poor ovarian responders (POR) or advanced-age patients; and (4) Metformin pretreatment, which demonstrates well-documented efficacy in improving ART outcomes for polycystic ovary syndrome (PCOS) patients, particularly recommended for those with glucose intolerance or insulin resistance prior to treatment. Regarding ovarian stimulation protocols, commonly utilized approaches include gonadotropin-releasing hormone agonist (GnRH-a) regimens, gonadotropin-releasing hormone antagonist (GnRH-A), protocols, and progestin-primed ovarian stimulation (PPOS). Gonadotropins (Gn) are classified into natural-derived preparations (human menopausal gonadotropin, HMG; urinary follicle-stimulating hormone, uFSH) and recombinant formulations (recombinant FSH, rFSH; recombinant LH, rLH; recombinant human chorionic gonadotropin, rhCG). Substantial evidence indicates that high-purity Gn facilitates precise follicular recruitment and enhances live birth rates, whereas low-purity alternatives may compromise pregnancy outcomes due to impurity interference (5). Nevertheless, clinical medication selection necessitates integrated consideration of multifactorial determinants, particularly patients’ socioeconomic constraints.

The Gn step-down protocol theoretically aims to recapitulate physiological FSH secretion patterns: initiating with higher doses to induce multifollicular recruitment, followed by progressive dose reduction as follicular numbers increase. This approach approximates natural ovulation cycles and may mitigate ovarian hyperstimulation syndrome (OHSS) risk (6). In contrast, the step-up protocol primarily targets specific populations (e.g., poor responders), enhancing follicular developmental synchrony. When unexpected suboptimal response occurs in anticipated normal responders, increasing Gn doses may improve clinical outcomes (7). Given substantial interindividual variability in ovarian response, clinical practice typically involves the first monitoring visit around stimulation day 4 to determine Gn dose adjustment necessity. However, ESHRE guidelines discourage routine Gn dose modifications to optimize COS outcomes. Current evidence indicates no significant benefits in efficacy (cumulative live birth rates) or safety (OHSS risk reduction) from such adjustments (8). The Italian Delphi Consensus further highlights ongoing controversy regarding whether Gn dose adjustments (e.g., ± 12.5 IU) confer significant improvements in COS outcomes. No agreement exists on the clinical relevance or appropriate magnitude of such modifications during ovarian stimulation. This consensus emphasizes that determining an optimal starting Gn dose remains paramount (9).

### 1.1 Novel conceptual framework

To address the fragmentation of existing knowledge and move beyond a purely descriptive inventory, we propose a three-layer integrative framework for conceptualizing and predicting ovarian response during COS.

Layer 1 – Baseline Ovarian Reserve comprises static markers (age, BMI, AMH, AFC, inhibin B, basal FSH/LH ratio) that define the pre-stimulation probability of poor, normal, or high response.

Layer 2 – Dynamic Response Efficiency includes intra-cycle indicators (FORT, FOI, OSI, ORPI, early/late FOI, modified FORT) that reflect the actual follicular response to exogenous gonadotropins and allow real-time adjustment.

Layer 3 – Molecular & Environmental Modulators encompasses genetic polymorphisms (e.g., FSHR, AMHR2, BMP15), epigenetic marks, and exposure to endocrine-disrupting chemicals, which explain inter-individual variability beyond clinical parameters.

This framework links pre-treatment assessment, ongoing cycle monitoring, and individual susceptibility factors, thereby providing a roadmap for personalized COS. The following sections systematically review each layer, and we subsequently propose a clinical decision algorithm that integrates these three layers into a coherent predictive model (Section 6).

### 1.2 Evidence grading and critical appraisal

Although this is a narrative review, we have applied a structured approach to evaluate the quality of evidence. We classified studies as high level (systematic reviews/meta-analyses and large RCTs), moderate level (prospective cohort studies and well-designed case-control studies), and low level (retrospective studies, case series, and small cross-sectional studies). For each indicator, we explicitly discuss limitations such as small sample size, heterogeneity in COS protocols, lack of blinding, and potential publication bias. This critical appraisal is summarized in **Table 1**. Readers should interpret the findings with these evidence grades in mind.

**Table 1:** Evidence grading and clinical recommendation of ovarian response markers.

Marker/index	Evidence level	Main study types	Limitations	Clinical recommendation strength
Age, AMH, AFC	High	Meta-analyses, RCTs, large cohorts	AFC operator-dependent; AMH assay variability; high heterogeneity	Strong
BMI	Moderate	Prospective cohorts	Confounding by PCOS	Moderate
FSH/LH ratio, inhibin B	Moderate to Low	Prospective/retrospective cohorts	Cycle dependence; limited meta-analysis	Moderate
FORT, FOI	Low to Moderate	Retrospective cohorts, reviews	No large-scale meta-analysis; variable cutoffs	Moderate
OSI, ORPI	Moderate to Low	Single-center retrospective	Protocol-dependent; limited RCT data	Moderate
Early FORT, MOSI	Low	Small prospective studies	Lack external validation	Weak
Genetic polymorphisms	Low	Candidate gene studies	Population heterogeneity; small samples	Weak
3D ultrasound indices	Low	Single-center trials	Equipment-dependent	Weak
Multi-omics signatures	Very low	Exploratory studies	Not clinically available	Weak

Evidence levels are based on study design and quality: High (meta-analyses/RCTs), Moderate (prospective cohorts), Low (retrospective/small studies), Very low (exploratory). Recommendation strength integrates evidence level, effect size, and clinical utility. See abbreviations list.

## 2 Baseline predictors of ovarian response

### 2.1 Age

Female fertility declines progressively with age, with significant reduction after 35 years and accelerated deterioration after 38 years (10). Age is a risk factor for diminished ovarian reserve, poor response, and slow follicular development. Its predictive value is limited in women under 30 years (due to stable reserve) but effective in those over 38 years (11). Significant heterogeneity in reproductive potential exists among same-aged women, necessitating multi-marker assessment. The POSEIDON criteria (2016) classify patients into four groups based on age, ovarian reserve markers (AFC, AMH), and ovarian response, enabling tailored protocols (12). Although age is a well−established non−modifiable predictor, its limited sensitivity in younger women underscores the need for additional biomarkers to improve individualized risk stratification.

### 2.2 Body mass index

Elevated BMI is associated with ovulatory dysfunction, impaired endometrial receptivity, reduced oocyte/embryo quality, and lower ART success rates (13). Obese patients require higher gonadotropin doses, longer stimulation, and have fewer oocytes retrieved (14, 15). The risk of anovulatory infertility increases with BMI (16). In PCOS patients, obesity independently predicts poorer IVF outcomes, including lower live birth rates and higher miscarriage risks (17). However, the association between obesity and adverse outcomes may diminish with increasing maternal age. Most available studies are retrospective and potentially confounded by PCOS; prospective studies that adequately control for PCOS status are needed to clarify the independent effect of BMI on ovarian response.

### 2.3 Conventional ovarian reserve and predictive markers

#### 2.3.1 Anti-Müllerian hormone

AMH, secreted by granulosa cells of preantral/small antral follicles, correlates positively with follicle count, declines with age, and remains stable across the menstrual cycle, establishing it as a reliable ovarian reserve marker (18). AMH predicts ovarian response intensity, guiding Gn dose and protocol selection, but does not improve ongoing pregnancy rates (19). AMH primarily reflects functional ovarian reserve (growing follicle pool) rather than the primordial pool, especially in young women (20, 21). AMH levels are influenced by hormonal contraceptives, obesity, and vitamin D. AMH has higher predictive value for menopause timing in women >45 years but limited sensitivity for early menopause in younger women; serial monitoring may improve accuracy (22). No single marker can independently assess both reserve and response, necessitating integrated assessment (23). The evidence supporting AMH is strong for predicting oocyte yield but only moderate for improving live birth rates. Moreover, the lack of assay standardization and the considerable between−study heterogeneity (I^2^ = 95.65%) limit the comparability of results across different studies.

#### 2.3.2 Antral follicle count

AFC, measured as total follicles (2–10 mm) in both ovaries via transvaginal ultrasound, objectively reflects ovarian reserve (24) and has strong predictive value for IVF outcomes (25). AMH and AFC are generally concordant, but discordance occurs in some women (26). AFC is operator-dependent and influenced by ultrasound resolution, ovarian position, and obscuring pathologies (e.g., endometriomas) (26). AMH declines more rapidly with age than AFC, and the two markers reflect different biological characteristics of antral follicles (27, 28). AFC is superior for predicting poor ovarian response (<6 oocytes), whereas AMH is better for high response (>18 oocytes); combining both with age optimizes predictive accuracy (28–30). Both markers also partially indicate oocyte quality (31, 32). The evidence level for AFC is high for predicting poor ovarian response but only moderate for high response. Operator dependency and the lack of universal standardization remain important limitations that affect its reproducibility across different centers.

#### 2.3.3 Follicle-stimulating hormone/luteinizing hormone ratio

Basal FSH alone cannot reliably assess ovarian responsiveness (33). An elevated basal FSH/LH ratio (≥3.5) is associated with poor ovarian response, higher Gn requirements, and lower oocyte yield (34). A ratio >3 increases cycle cancellation rates (35). Predictive value is age-dependent: it predicts outcomes in women ≥35 years but not in younger women (36). Controversy remains, as some studies find no independent association with live birth rate (37). In antagonist protocols, an elevated basal ratio predicts poor response (≤5 oocytes) and reduced embryo formation, while an elevated ratio on trigger day correlates with increased oocyte yield but is associated with elevated progesterone and endometrial thinning, leading to decreased pregnancy rates. Arat et al. (37) reported that FSH/LH ratio ≥2 is associated with reduced oocyte yield but no independent association with live birth rate. Current evidence for the FSH/LH ratio is moderate to low, and prospective large−scale studies with standardized testing protocols are required to establish clinically useful thresholds. As a supplementary marker, it may be considered when core markers are equivocal or in specific age groups.

#### 2.3.4 Inhibin B

Inhibin B, a heterodimeric glycoprotein secreted by granulosa cells of developing antral follicles (38), correlates positively with AMH/AFC and negatively with FSH (39). It predicts oocyte yield, particularly in women with diminished ovarian reserve (40). However, a recent prospective study in expected poor responders (AMH <1.1 ng/mL) found that inhibin B does not significantly improve predictive accuracy beyond AMH and AFC, suggesting a complementary rather than replacement role (41). Dynamic changes in inhibin B during stimulation correlate with oocyte yield and may guide Gn dosing, with thresholds of 295.5 ng/L and 515.5 ng/L for predicting poor and high response, respectively (42). Thus, inhibin B may be integrated into a multi-marker panel when AMH results are discordant or unreliable. Evidence for inhibin B is moderate; its clinical utility is limited as it adds little value to AMH and AFC. It may be reserved for cases where core marker results are discordant or unreliable.

## 3 Dynamic indicators derived during controlled ovarian stimulation

However, it should be noted that the aforementioned markers provide only static assessment parameters of ovarian reserve and fail to fully capture the dynamic biological processes of follicular development under exogenous ovarian stimulation. Given the complexity of ovarian response prediction and the limitations of single indicators in forecasting ovarian responsiveness, many researchers have developed multivariate models that integrate multiple indicators to enhance the sensitivity and specificity of predictive accuracy.

### 3.1 Follicular output rate

In 2011, Genro et al. first introduced the concept of follicular output rate (FORT) in a study of agonist-controlled ovarian stimulation protocols (43). FORT is defined as the ratio of preovulatory follicles (16–22 mm in diameter) on the day of hCG administration to antral follicles (3–8 mm) at stimulation initiation. A low FORT indicates a reduced ratio of mature follicles to the baseline antral follicle cohort after ovarian stimulation, establishing it as an intuitive indicator of ovarian responsiveness. Notably, decreased FORT shows no direct correlation with ovarian reserve, meaning patients may exhibit low FORT despite normal reserve. Similarly, using agonist protocols, Gallot et al. observed a progressive increase in oocytes and embryos retrieved from low to high FORT groups (p<0.001), a finding (44) corroborated by Hassan et al. Zhang et al. defined low (FORT < 0.5), medium (FORT 0.5-0.73), and high FORT (FORT > 0.73) groups using tertile values. The study found that in non-PCOS patients, higher FORT levels were significantly associated with increased oocyte yield, number of transferable embryos, high-quality embryo rate, embryo implantation rate, and clinical pregnancy rate. FORT serves as an indicator of ovarian response to FSH and reflects follicular health and reproductive potential. However, in PCOS patients, while the high-FORT group yielded the most oocytes, the medium-FORT group demonstrated significantly higher fertilization rates (69.49%) and high-quality embryo rates (72.71%) compared to both low- and high-FORT groups. A medium FORT value may indicate better outcomes, whereas excessively high FORT could lead to follicular hyper-response and is associated with diminished oocyte quality. This phenomenon is likely due to PCOS-specific pathophysiological influences, such as the hyperestrogenic environment (40).

Grynberg et al. discussed the potential utility of FORT as a quantitative and qualitative marker of ovarian responsiveness to gonadotropins and its possible implications for the applicability of the POSEIDON criteria (12, 45). They noted that the POSEIDON classification subdivides low-prognosis patients into four groups. Specifically, Group 1 and Group 2 patients exhibit normal ovarian reserve (AFC > 5, AMH > 1.2 ng/mL) yet demonstrate a poor response to FSH stimulation, requiring longer stimulation duration and/or higher FSH doses. This indicates that traditional markers (AFC and AMH) inadequately predict ovarian response. The study also found that FORT was negatively correlated with AMH, and higher FORT levels were significantly associated with improved ART outcomes (oocyte yield, embryo quality, pregnancy rate). These findings suggest that FORT aids in identifying poor responders and guides individualized treatment, thereby optimizing stimulation protocols and prognosis for patients across POSEIDON subgroups. Furthermore, Adela et al. conducted a prospective study of 215 women comparing the ability of computer-automated (46), volume-based follicular output rate (FORT-V) derived from 3D ultrasound follicular volume measurements and manually measured, diameter-based follicular output rate (FORT-D) to predict the number of mature oocytes. The results showed that FORT-V consistently outperformed FORT-D. This also suggests that in-depth analysis of computer-generated antral follicle characterization data may aid in evaluating ovarian stimulation protocols.

In conclusion, there is still a lack of effective tools for accurately assessing ovarian responsiveness. FORT has been demonstrated to be a relevant and critical quantitative and qualitative indicator for use in ART (45). However, several limitations exist in the application of FORT. Its assessment of FSH responsiveness is based on follicles measuring 16–22 mm on the day of hCG administration. Yet, small follicles undetected by baseline ultrasound may mature to intermediate sizes during COS under Gn drive, potentially biasing the results. Additionally, current technology cannot track the development of individual follicles, preventing the assessment of potential differential growth among follicles driven by Gn within the same cycle (43). Research has identified stimulation day 5 as the optimal early timepoint for predicting the timing of trigger and the risk of OHSS. Some scholars have proposed the “early Follicular Output Rate (early FORT)” as a new metric for evaluating ovarian stimulation efficacy. It is defined as the ratio of the number of follicles **≥**10–11 mm in diameter on day 5/6 of ovarian stimulation to the baseline AFC. The researchers (47) suggest that early assessment of ovarian response might facilitate individualized dose adjustments, although its clinical value remains undefined and warrants further investigation. Moreover, other researchers have proposed the “modified Follicular Output Rate (modified FORT)”, defined as the ratio of the number of follicles **≥**12 mm on trigger day to the baseline AFC. This metric may assess the potential of smaller follicles to develop into mature oocytes. However, modified FORT requires further study in different female subpopulations to establish specific reference ranges.

### 3.2 Follicle-To-Oocyte Index

Alviggi et al. (48) evaluated ovarian response to exogenous Gn in ART patients using the Follicle-To-Oocyte Index (FOI), proposing it as a significant predictor of ovarian sensitivity, where FOI is defined as the ratio of oocytes retrieved to the AFC at stimulation initiation. This metric specifically highlights inefficient ovarian reserve utilization when actual oocyte yield falls below baseline AFC expectations. Results demonstrated FOI’s superiority over traditional ovarian reserve markers in reflecting follicular response to exogenous gonadotropins and growth dynamics. Furthermore, FOI serves as a key parameter for assessing impaired ovarian response (poor ovarian response), with an FOI > 50% indicating normal ovarian sensitivity and an FOI ≤ 50% signifying suboptimal COS outcomes. Furthermore, FOI is proposed as a key parameter for assessing impaired ovarian response to exogenous Gn stimulation (i.e., poor ovarian response). Researchers differentiated suboptimal response—defined by a low oocyte yield (4–9 oocytes) with preserved response efficiency when FOI exceeds 50%, indicating inherently diminished ovarian reserve—from hypo-response, characterized by oocyte retrieval substantially below AFC-predicted levels (FOI ≤50%), reflecting ineffective reserve utilization that may coexist with poor response. The concurrent presentation of poor response and hypo-response denotes both low baseline reserve and compromised efficiency. In a cohort of 243 Vietnamese low-prognosis infertile women categorized under POSEIDON criteria, FOI consistently demonstrated positive correlation with actual oocyte yield across all subgroups (49). Crucially, no significant associations were observed between FOI values and age, BMI, AMH levels, or total FSH dose, establishing FOI as an independent and effective indicator of ovarian sensitivity. These attributes position FOI as a predictive marker for ovarian responsiveness, particularly valuable for identifying patients with reduced ovarian sensitivity and guiding personalized treatment strategies in low-prognosis populations (50). Notably, Bessow et al. revealed that women with numerous small antral follicles (AFC < 6) exhibited reduced responsiveness to FSH stimulation within this follicular cohort. This finding underscores that ovarian reserve assessment and responsiveness prediction should incorporate not only AFC but also follicular size distribution, particularly the proportion of small follicles.

Retrospective analysis by Li et al. identified FOI as an effective predictor of ovarian responsiveness and pregnancy outcomes following single embryo transfer (51). Another retrospective study of 264 IVF cycles further demonstrated that elevated FOI primarily correlated positively with live birth rates after the first transfer (52). FOI analysis may yield divergent results in older women. The predictive value of AFC is limited in this population, potentially attributable to mitochondrial dysfunction, increased granulosa cell apoptosis, and elevated oxidative stress in follicular fluid. The underlying mechanism may involve upregulated miR-484 expression in granulosa cells, which impairs mitochondrial function and induces apoptosis by targeting and suppressing YAP1, ultimately contributing to diminished ovarian reserve. FOI analysis may yield divergent results in older women. The predictive value of AFC is limited in this population, potentially attributable to mitochondrial dysfunction, increased granulosa cell apoptosis, and elevated oxidative stress in follicular fluid. The underlying mechanism may involve upregulated miR-484 expression in granulosa cells, which impairs mitochondrial function and induces apoptosis by targeting and suppressing YAP1, ultimately contributing to diminished ovarian reserve (53).

Based on a recent expert consensus (54), researchers proposed the “late follicular-oocyte index (late FOI)”, defined as the ratio of total oocytes retrieved to the AFC on day 5 of ovarian stimulation. This metric aims to minimize variability in AFC measurements across centers, cycles, and operators. The consensus recommends: **≥**80% as optimal, **≥**50% as the minimum acceptable threshold, and <50% indicating suboptimal outcomes. This index can be extended to the “mature follicular-oocyte index” (mature FOI), which is defined as the ratio of mature (MII-stage) oocytes retrieved to the antral follicle count (AFC) on day 5. This ratio serves as an indicator of stimulation and trigger efficiency. Recommended thresholds are **≥**90% (optimal) and **≥**75% (minimum). Values outside this range warrant investigation into factors affecting MII oocyte yield, as they ultimately impact reproductive outcomes. However, experts advise applying mature FOI exclusively to intracytoplasmic sperm injection (ICSI) cycles (47).

### 3.3 Ovarian sensitivity index

In 2011, Biasoni et al. introduced the Ovarian Sensitivity Index (OSI), establishing a quantitative relationship between oocyte yield and gonadotropin stimulation intensity (55). OSI is defined as the exogenous gonadotropin dosage required per oocyte retrieved (total dose/number of oocytes). Consequently, lower OSI values indicate higher ovarian sensitivity, and vice versa. OSI can serve as a partially independent parameter in predictive models designed to forecast ovarian response to exogenous gonadotropins. This metric effectively corrects for interference from ovarian stimulation medication dosage, providing a more objective reflection of the drug requirement per developing oocyte. It thus offers a basis for individualized COS protocol design and initial Gn dosage selection, demonstrating significant utility in comparative studies of different ovarian stimulation protocols. Some patients exhibit abnormally high or low responses to ovarian stimulation. Even with personalized dosage adjustments, their oocyte yield may deviate from ovarian reserve potential, failing to accurately reflect full ovarian capacity. This discrepancy arises because FSH receptor quantity and distribution patterns on follicles constitute the fundamental basis for follicular response to exogenous Gn, co-regulated with individual follicular wave dynamics during recruitment. Substantial interpatient variations exist in FSH receptor characteristics across follicular surfaces (56). Full ovarian potential might only be expressed under maximal Gn stimulation, yet clinical safety constraints limit maximum dosage application. Consequently, the true ovarian responsiveness to gonadotropins cannot be precisely determined under current therapeutic boundaries.

In 2014, Li et al. (56) demonstrated that the OSI is a more reliable indicator of ovarian response to gonadotropin stimulation than the number of oocytes retrieved. Their research confirmed significant positive correlations between OSI and established parameters within ovarian response assessment systems—including ovarian stimulation duration, total gonadotropin dose, peak serum estradiol concentration, and number of follicles 16 mm—demonstrating OSI’s clinical validity. The authors propose OSI as a ≥synergistic complement to the number of oocytes retrieved in COS research. Substantiating this value, Selcuk et al. (57) found OSI significantly outperformed traditional biomarkers (AMH, AFC) in predicting both poor and high ovarian response. Further mechanistic insights from Revelli et al. reveal that OSI, which intrinsically incorporates ovarian response efficiency and stimulation intensity, exhibits negative correlations with age and BMI while showing positive correlations with AMH and AFC. Revelli et al. demonstrated that the OSI integrates ovarian response efficiency with pharmacological stimulation intensity (58). OSI exhibits a negative correlation with patient age and BMI but a positive correlation with AMH levels and AFC. It independently predicts clinical pregnancy rates more effectively than oocyte yield alone. Furthermore, OSI demonstrates high inter-cycle consistency (82%) when the same patient switches between different controlled ovarian stimulation protocols, specifically GnRH agonist versus antagonist regimens. This consistency indicates that OSI primarily reflects inherent patient ovarian characteristics rather than the influence of a specific stimulation protocol. Consequently, OSI is applicable for developing individualized predictive models of ovarian response and pregnancy outcomes in IVF. Huber et al. (59) classified IVF patients into poor, normal, and high responder categories using standard statistical methods, defining poor responders as those with an OSI below 1.697 per IU and high responders as those exceeding 10.07 per IU. Their analysis revealed distinct clinical profiles: the poor responder group was significantly older, required the highest total FSH dose, and produced the lowest oocyte yield. The normal responder group exhibited intermediate age, FSH dosage requirements, and oocyte retrieval numbers. Conversely, the high responder group was youngest, required the lowest total FSH dose, and achieved the highest oocyte yield. Clinical pregnancy rates demonstrated a positive correlation with both oocyte yield and the OSI. However, contrasting research (60) found no significant difference in pregnancy rates between a high-dose gonadotropin group, characterized by low OSI and oocyte yields of at least 16, and a low-dose group, characterized by high OSI and oocyte yields below 6. A separate retrospective analysis further confirmed that OSI independently predicts the likelihood of live birth in patients of advanced maternal age with poor prognoses. This highlights OSI’s capacity to address a key limitation of traditional ovarian reserve markers, such as AFC, which primarily reflect quantitative aspects while overlooking oocyte quality. Consequently, OSI emerges as a valuable new tool for individualized counseling in patients with poor prognosis, particularly those of advanced maternal age or with premature ovarian insufficiency (61).

For patients with at least one prior IVF cycle, the OSI is an easily measurable parameter. Its straightforward calculation and low cost position OSI as an economical alternative to AMH, particularly suitable for optimizing individualized ovarian stimulation strategies in resource-limited settings (55). However, OSI exhibits several limitations. First, it can only be calculated after completing the initial IVF cycle and thus cannot predict outcomes prior to the first treatment cycle. Second, OSI does not account for variations in GnRH analogue type, gonadotropin formulation, starting gonadotropin dose, or specific stimulation protocols (48).

In 2020, Camargo et al. proposed the Modified Ovarian Sensitivity Index (MOSI), defined as the ratio of the number of follicles ≥6 mm measured on stimulation day 3 or 4 to the initial FSH dose, multiplied by 1000. In their study, MOSI was applied for early prediction in IVF cycles utilizing antagonist protocols. Subsequent retrospective and prospective investigations confirmed that MOSI independently predicts the number of high-quality embryos, blastocyst formation potential, and pregnancy likelihood. It also demonstrated significant correlations with oocyte yield and the number of metaphase II (MII) oocytes. With a predictive accuracy ranging from 61.4% to 68.5%, MOSI provides a basis for early clinical decision-making, although further validation of its applicability across specific patient subgroups is warranted (62). Future research should prioritize elucidating the molecular mechanisms governing ovarian response to gonadotropins and systematically evaluating the impact of different ovarian stimulation protocols on treatment outcomes in poor responders. Such efforts would establish a stronger theoretical foundation for clinical management strategies.

### 3.4 Ovarian response prediction index

Joao et al. (63) first proposed a novel ovarian response evaluation metric—the Ovarian Response Prediction Index (ORPI). This index is based on prior observations demonstrating that ovarian response to stimulation correlates positively with AMH levels and AFC (2–9 mm), but negatively with patient age. The ORPI is calculated using the formula: ORPI = AMH (ng/ml) **×** AFC (2–9 mm)/Age (years). The study included 101 patients undergoing intracytoplasmic sperm injection (ICSI). Results demonstrated significant positive correlations between ORPI and both the total number of oocytes retrieved and the total number of mature (MII) oocytes. ORPI also significantly correlated with pregnancy rates. The index exhibited excellent predictive power for poor ovarian response (AUC:0.84) and demonstrated good predictive ability for retrieving ≥4 MII oocytes (AUC: 0.89) and for excessive ovarian response (AUC: 0.89). Furthermore, calculated ORPI values in the study were not affected by the choice of ovarian stimulation protocol (agonist or antagonist) or gonadotropin dosage (64). An additional advantage of ORPI is its capacity to estimate ovarian response prior to the initiation of any treatment.

In a retrospective analysis of 521 infertile women undergoing IVF/ICSI treatment, Sun et al. demonstrated that the ORPI, AMH, and AMH/age ratio exhibited significant predictive efficacy for the number of oocytes retrieved (65). Zhou et al. found that ORPI demonstrated superior performance in predicting hyper-response (>15 follicles in a cohort of 15 patients, including 7 OHSS cases), whereas the AFC/age ratio was identified as the optimal indicator for assessing ovarian reserve and predicting suboptimal response (66). Carla et al. enrolled 188 infertile women aged <38 years undergoing their first IVF/ICSI cycle and evaluated the predictive power of age, FSH, AMH, AFC, and ORPI using ROC analysis (67). The results showed that for predicting ovarian hyper-response, ORPI demonstrated the strongest predictive power, outperforming all other individual markers and combined models. However, neither individual nor combined ovarian reserve tests (including ORPI) showed adequate predictive ability for poor response. This study highlights the value of integrating multi-parameter indices (e.g., ORPI) to improve hyper-response prediction, but underscores the need to explore novel approaches for poor response prediction.

However, in a prospective analysis of 550 treatment-naïve IVF/ICSI patients with unexplained infertility, Mahnaz et al. demonstrated that AFC achieved superior predictive efficacy for both poor ovarian response (AUC = 0.85) and hyper-response (AUC = 0.79) compared to AMH and the ORPI, with ORPI providing no incremental predictive value (68). AMH, AFC, and ORPI all exhibited poor predictive capacity for clinical pregnancy and live birth rates (AUC <0.60). Nevertheless, the study has inherent limitations: the findings may lack generalizability to infertility populations with other etiologies, and the exclusion of women of advanced maternal age (>39 years) precluded comprehensive assessment of age-related impacts on pregnancy outcomes.

### 3.5 Initial gonadotropin dose

Current research confirms a correlation between the initial Gn dose and ovarian responsiveness. While elevated Gn doses generally increase oocyte yield, ovarian response demonstrates complex nonlinear dynamics. Contemporary consensus posits that COS should target optimal rather than maximal oocyte retrieval (58). In antagonist protocols, evidence-based Gn starting doses are stratified by predicted response categories: hyper-responders (HOR) receive 100–150 IU, normo-responders (NOR) 150–225 IU, and diminished/poor ovarian responders (DOR/POR) 225–300 IU (69). For repeat IVF cycles, dosing adjustments derive from historical response patterns, whereas treatment-naive patients require empirical dosing integrating baseline parameters (AMH, AFC, age, BMI). This experience-dependent paradigm lacks quantitative standardization, introducing clinician-specific variability that may compromise therapeutic precision. Consequently, initial Gn dosing–a critical determinant of IVF success–remains predominantly subjective. Although international reproductive societies (ESHRE, ASRM) have proposed guidelines, universally accepted protocols remain unrealized (3).

### 3.6 Comparative synthesis of dynamic indices: FORT, FOI, OSI, and ORPI

The four dynamic indices share the goal of improving ovarian response prediction beyond static markers, yet they differ substantially in their inputs, outputs, and clinical positioning. **Table 2** provides a direct comparison.

**Table 2:** Comparative analysis of four core dynamic ovarian response indices.

Index	Definition/formula	Primary prediction target	Strengths	Limitations	Optimal clinical population
FORT	Preovulatory follicles/baseline AFC	Follicular maturation efficiency	Non-invasive; ultrasound-based	Not directly associated with oocyte yield	Non-PCOS; pregnancy prediction
FOI	Retrieved oocytes/baseline AFC	Ovarian sensitivity to Gn	Directly reflects oocyte retrieval efficiency	Affected by AFC measurement error	POSEIDON low-prognosis patients
OSI	Total Gn dose/number of oocytes	Ovarian sensitivity to medication	Corrects Gn dosage interference	Calculated post-retrieval only	Repeated IVF cycles; advanced age
ORPI	AMH × AFC/Age	Pre-stimulation response prediction	Pre-cycle evaluation; protocol-independent	Poor performance for POR prediction	First IVF cycle; HOR screening

FORT, preovulatory follicles/baseline AFC; FOI, oocytes/baseline AFC; OSI, total Gn dose/oocytes; ORPI, (AMH × AFC)/age. See text for clinical applications.

FORT focuses on the proportion of antral follicles that reach pre-ovulatory size. Its strength lies in reflecting follicular health and maturation efficiency, and it has been positively associated with embryo quality and pregnancy in non-PCOS patients. However, its reliance on diameter thresholds (16-22 mm) and inability to track individual follicles introduce bias. Moreover, its predictive value in PCOS patients is inverted, with medium FORT yielding better outcomes than high FORT.

FOI measures oocyte yield per baseline antral follicle, thereby capturing ovarian sensitivity independent of age, BMI, and FSH dose. A FOI > 50% defines normal sensitivity. Its main limitation is reduced predictive power in women over 38 years, likely due to mitochondrial dysfunction and granulosa cell apoptosis. The recently proposed late FOI (using day-5 AFC) improves inter-operator consistency.

OSI (total Gn dose per oocyte) is unique in accounting for stimulation intensity. It has high inter-cycle consistency (82% when switching between agonist and antagonist protocols) and correlates with live birth rates. However, OSI cannot be calculated before the first cycle, making it unsuitable for initial prediction. It is most valuable for repeat cycles and for comparing protocol efficiency.

ORPI (AMH × AFC / age) is the only pre-treatment composite index. It has shown excellent predictive power for hyper-response (AUC 0.89) in some studies, but others found no added value over AFC alone. Its performance is highly population-dependent, and it does not incorporate dynamic changes during stimulation.

The clinical utility of the four dynamic indices differs according to the phase and objective of assessment. Prior to stimulation, ORPI can be employed as a predictive tool, particularly for hyper-response screening, although its incremental value relative to AFC alone remains a subject of debate. During the stimulation phase, FORT and early FORT enable real-time monitoring of follicular responsiveness. For the evaluation of ovarian sensitivity to exogenous gonadotropins, FOI and OSI represent the most reliable indicators. In the context of repeated ART cycles, OSI demonstrates superior stability across cycles and is cost-effective, making it a practical choice for longitudinal management.

## 4 Genetic and environmental modulators of ovarian response

### 4.1 Genetic polymorphisms

Existing research confirms that genetic regulation of ovarian response involves both monogenic and polygenic mechanisms. POI and DOR are associated with mutations in DNA damage repair and meiosis-related genes (70, 71), while PCOS is influenced by polygenic regulation involving pathways such as insulin signaling and androgen metabolism (72). Genome-wide association studies suggest that the age of natural menopause may be closely associated with DNA damage repair and hypothalamic signaling genes (73), while impaired oocyte maturation is linked to specific gene mutations (74–76) (**Table 3**).

**Table 3:** Ovarian response-related gene regulation.

Dysfunction categories	Critical genes	Pathophysiological mechanisms
POI and DOR	POI/DOR Susceptibility Genes (e.g., *STAG3, NOBOX, FSHR*)	Impairs meiotic chromosome segregation, follicular development, or hormone signaling by disrupting the cohesin complex via STAG3, leading to oocyte depletion or dysfunction.
PCOS	*LHCGR(2p16.3), THADA(2p21)and DENND1A(9q33.3)*	Induces pathogenesis through sex hormone dysregulation, ovulatory disorders, and insulin resistance
Alterations in Natural Menopausal Age	DNA Damage Repair (DDR) Pathway Genes(e.g., *BRCA1, CHEK2, HELB*); Hypothalamic Signaling Genes (e.g., *KISS1R, TAC3*)	DDR pathway genes influence ovarian reserve depletion by maintaining oocyte genomic stability, while hypothalamic signaling genes regulate the reproductive hormone axis. Together, they synergistically determine the rate of follicular pool attrition, thereby governing the timing of natural menopause.
Oocyte Maturation Arrest	*TUBB8, PATL2, WEE2, ZP1, ZP2, ZP3*, and related genes	PATL2 mutations cause oocyte maturation arrest at the GV stage; *TUBB8* mutations induce spindle malformation, resulting in MI-stage arrest; *WEE2* mutations lead to fertilization failure (characterized by absent pronuclear formation); *ZP1, ZP2*, and *ZP3* mutations disrupt zona pellucida architecture, impairing sperm binding.

This table offers an overview of research into gene regulation linked to ovarian responsiveness.

Studies (77) have focused on women classified as POSEIDON Groups 1 and 2, who exhibit unexpected poor response (<4 oocytes retrieved) or suboptimal response (4–9 oocytes) to Gn stimulation despite normal ovarian reserve parameters (AFC ≥5 or AMH ≥1.2 ng/mL). The underlying pathological mechanism may involve polygenic variations in gonadotropins and their receptors. Specifically, the Ser/Ser genotype of the FSHR Asn680Ser (rs6166) polymorphism correlates with reduced FSH sensitivity, requiring higher medication doses and resulting in fewer oocytes. Mutations in the luteinizing hormone subunit beta gene (LHB) (rs1800447, rs34349826) may decrease LH activity, necessitating adjustments to FSH/LH protocols. Conversely, the C/C genotype of ESR1 -397T>C (rs2234693) is associated with a more favorable ovarian response. In addition, genetic variants in AMH/AMHR2, CYP19A1, MTHFR, and BMP15/GDF9 have been linked to ovarian response, oocyte maturation, or risk of OHSS.

Recent studies have further demonstrated that FORT and FOI are regulated by the synergistic effects of multiple genetic variants. Among women with normal ovarian reserve, non-FSHR gene variants play a significant role: AMHR2 rs2002555 (G/G) may reduce FORT by enhancing AMH signaling and thereby decreasing follicle sensitivity to FSH; COMT rs4680 (A/A) may increase FORT by slowing estrogen metabolism; and BMP15 rs3897937 (G/G) is associated with reduced FOI (78). In women with diminished ovarian reserve (DOR), the A allele of FSHR rs6165 and rs6166 is associated with improved FORT and FOI, independent of baseline ovarian reserve markers (79). Further analyses indicate an additive effect of FSHR diplotypes, with the AA/GG combination of rs6166 and rs1394205 (homozygous A/A for rs6166 and G/G for rs1394205) being closely associated with higher FOI and oocyte yield, suggesting that this diplotype may serve as a potential genetic marker for predicting ovarian response sensitivity (80).

Research highlights that ovarian response is coordinately regulated by multiple genes. Emerging pharmacoepigenomic approaches, such as miRNA expression profiles, show potential for refining ovarian response prediction. Pre-cycle screening of key genetic loci can guide personalized stimulation protocols, maximizing efficacy (e.g., oocyte yield, pregnancy rates) while minimizing complications (e.g., OHSS). This advancement signifies a new era of precision medicine in IVF.

### 4.2 Environmental and occupational exposures

Environmental exposures represent significant risk factors for female infertility (81). Multiple exogenous agents can impair reproductive health through distinct mechanisms: air pollutants (polycyclic aromatic hydrocarbons, heavy metals, and nitrogen oxides) impair follicular development, ovarian function, and embryo quality by inducing oxidative stress, DNA damage, and epigenetic alterations, consequently reducing success rates in assisted reproductive technology; noise pollution activates the hypothalamic−pituitary−adrenal axis, elevating cortisol levels and indirectly disrupting reproductive endocrine homeostasis; bioaccumulative heavy metals such as lead, cadmium, and mercury demonstrate significant associations with spontaneous abortion, preterm birth, and fetal growth restriction; occupational exposure to pesticides, industrial solvents (e.g., benzene, xylene), and endocrine−disrupting chemicals (EDCs) including bisphenol A and phthalates interferes with hormone synthesis, receptor binding, and ovarian cyclicity, leading to ovulatory disorders and diminished fertility; ionizing radiation directly damages germ cells, increasing risks of implantation failure and congenital anomalies (81).

EDCs are defined as natural or synthetic substances that disrupt endocrine function through hormone mimicry, signal interference, or receptor blockade, and they adversely impact reproductive health (82). Major EDCs include bisphenols (BPA, BPAF), heavy metals, pesticides, plasticizers, and physical factors such as tobacco smoke and PM2.5 particulate matter. These agents trigger pathological mechanisms including follicular developmental impairment (83), steroid synthesis inhibition (84), oxidative stress with DNA damage (85), epigenetic dysregulation (86), and gut microbiota−ovarian axis disruption (87). Collectively, these processes establish vicious cycles that contribute to polycystic ovary syndrome (PCOS), endometriosis, and recurrent pregnancy loss (88) (**Table 4**).

**Table 4:** EDCs pose a threat to female reproductive health.

Dysfunction categories	Pollutant categories	Biological effects
Ovarian Follicular Dysfunction	EDCs (e.g., Bisphenol A, 2,3,7,8-Tetrachlorodibenzo-p-dioxin) and Heavy Metals	Inhibits folliculogenesis-related gene expression, increases the proportion of atretic follicles, and reduces the number of antral follicles
Steroid Synthesis Inhibition	Plasticizers and Related Compounds	Downregulates steroidogenic enzyme genes, dysregulates the estrogen-progesterone equilibrium, and causes hormone synthesis dysregulation
Oxidative Stress and DNA Damage	Tobacco Smoke, PM2.5 and Bisphenol AF	Induces reactive oxygen species accumulation, leading to mitochondrial dysfunction and DNA damage, thereby reducing oocyte maturation rates
Epigenetic Aberrations	BPA and Other EDCs	Alters histone modifications (e.g., methylation), reprograms DNA methylation, and remodels non-coding RNA expression, thereby disrupting oocyte maturation-critical genes and potentially mediating transgenerational reproductive toxicity
Gut Microbiota-Ovarian Axis Disruption	Heavy Metals and Plasticizers	Alters gut microbiota composition, thereby disrupting estrogen enterohepatic recirculation and inducing endocrine disorders such as PCOS

This table presents a systematic summary of the existing body of evidence regarding the threats that Endocrine-Disrupting Chemicals (EDCs) pose to female reproductive health.

Ovarian response is closely associated with various exogenous exposure factors. Bisphenol A, phthalates, and similar compounds can disrupt the hormonal homeostasis of the hypothalamic−pituitary−ovarian axis, thereby adversely affecting follicle recruitment, development, morphology, and the spindle/chromosome alignment of oocytes (89). Tobacco−derived polycyclic aromatic hydrocarbons induce an imbalance between oxidative and antioxidant systems in granulosa cells (90). In addition, animal studies have demonstrated that exposure to cigarette smoke activates the Atg (autophagy−related genes) autophagy pathway, leading to follicle loss and consequently reduced ovarian response. Furthermore, women with long−term exposure to organic solvents, heavy metals such as lead and mercury, or pesticides also exhibit a decline in ovarian response (91). These substances induce excessive production of reactive oxygen species, directly compromising the DNA integrity of oocytes. Moreover, they promote ovarian granulosa cell apoptosis via oxidative stress, disrupt follicular microenvironment homeostasis, and ultimately reduce ovarian response (92).

Emerging evidence from recent systematic reviews and original studies has quantified the associations between exposure to EDCs and ovarian reserve parameters. A comprehensive systematic review by An et al. (93), analyzing 40 cohort studies on 20 pollutant types, demonstrated that perfluoroalkyl and polyfluoroalkyl substances (PFAS), phthalates (PAEs), polychlorinated biphenyls (PCBs), and particulate matter (PM2.5) are significantly associated with decreased anti−Müllerian hormone (AMH) and antral follicle count (AFC), particularly in women under 35 years of age. More specifically, Tian et al. (94) conducted a case−control study involving 182 women and found that serum concentrations of six disinfection by−products (DBPs) were significantly higher in patients with diminished ovarian reserve (DOR) compared to healthy controls. After covariate adjustment, all DBPs showed negative correlations with AMH and AFC and positive correlations with basal follicle−stimulating hormone (FSH), with the risk of DOR increasing as serum DBP concentrations increased. Similarly, a systematic review by Tzouma et al. (95), encompassing 14 observational studies, reported consistent associations between EDC exposure (including BPA, phthalates, and PFAS) and decreased ovarian reserve, as well as altered hormone levels (estradiol, luteinizing hormone, FSH).It is worth noting that not all studies have reported positive associations. Patel et al. (96), in a cohort of women with PCOS−related infertility, found no significant correlations between BPA or phthalate exposure and ovarian reserve markers.

Future research should target epigenetic biomarkers such as microRNAs to develop novel diagnostic and therapeutic approaches, and prioritize individualized genetic testing as well as mechanistic interventions against the impacts of environmental exposures (88).

## 5 Emerging techniques for ovarian response assessment

### 5.1 Advanced ultrasound parameters

Ultrasound assessment, particularly three-dimensional (3D) power Doppler angiography combined with virtual organ computer-aided analysis (VOCAL), has emerged as a valuable tool for predicting ovarian response to controlled ovarian stimulation. This technique enables quantitative evaluation of ovarian volume, antral follicle count (AFC), and vascular indices including the vascularization index (VI), flow index (FI), and vascularization-flow index (VFI), all of which demonstrate high intraobserver and interobserver reliability (intraclass correlation coefficients >0.985) (97).

A recent prospective study by Yang et al. introduced a novel 3D ultrasound parameter—follicular sphericity—and demonstrated that patients with lower sphericity (<0.716) achieved significantly higher oocyte retrieval rates, MII oocyte rates, follicular output rate (FORT), and ovarian sensitivity index (OSI) compared to those with higher sphericity, suggesting that follicular morphology reflects functional quality during controlled ovarian stimulation (98). Furthermore, Yin et al. evaluated ovarian reserve in patients with adenomyosis and endometriosis using 3D transvaginal ultrasound, finding that AFC, ovarian volume, VI, FI, and VFI were all significantly decreased in affected patients compared to healthy controls, with moderate to strong positive correlations between AMH and these 3D ultrasound parameters (r = 0.50, 0.48, 0.45 for VI, FI, and VFI, respectively; all P < 0.01) (99). Collectively, these findings support the integration of advanced ultrasound parameters—particularly 3D-derived AFC, ovarian volume, and vascular indices—into a multi-modal assessment framework to optimize prediction of ovarian response and personalize controlled ovarian stimulation protocols.

### 5.2 Multi-omics approaches

The follicular fluid (FF) microenvironment serves as a critical “black box” in determining ovarian response, and many OMICS approaches have begun to elucidate its molecular complexity. Proteomic studies have identified key FF proteins such as IGFBP5, LAMP2, and CDH5 that correlate with oocyte quality and embryo outcomes in PCOS patients (100). Transcriptomic analyses have revealed that the TGF-β signaling pathway in granulosa cells is associated with embryo quality in poor ovarian responders (101), and that extracellular vesicles in FF carry distinct non-coding RNA profiles capable of regulating granulosa cell function (102). Epigenetic research further demonstrates that DNA methylation age acceleration in FF predicts ovarian response parameters including peak estradiol levels and oocyte yield (103). Integrative multi-omics studies combining microbiome, metabolomic, and inflammatory protein profiling have provided additional insights into the complex interactions within the FF microenvironment (104, 105). Collectively, these findings support the concept that integrating proteomic, transcriptomic, genomic, and epigenomic data is essential for decoding the follicular fluid “black box” and its role in ovarian response.

## 6 Integrated clinical framework for ovarian response prediction

To address interindividual heterogeneity in ovarian response, we propose an Integrated Multi-dimensional Ovarian Response Prediction (IMORP) framework that synthesizes static reserve, dynamic responsiveness, genetic susceptibility, and environmental exposure into a hierarchical model for personalized controlled ovarian stimulation (COS).

Tier 1 (static baseline stratification, pre-stimulation) uses core indicators including age, BMI, AMH, AFC, basal FSH/LH ratio, and inhibin B to stratify patients into poor, normal, or high response categories, guiding initial gonadotropin dose and protocol selection.

Tier 2 (dynamic responsiveness monitoring, stimulation phase) employs FORT, FOI, OSI, ORPI, early FORT, and modified FORT to evaluate real-time follicular maturation efficiency and Gn sensitivity, informing dose adjustment, trigger timing, and OHSS risk assessment.

Tier 3 (genetic polymorphism correction) incorporates variants such as FSHR (rs6165, rs6166), AMHR2, ESR1, LHB, and BMP15 to refine Gn dosing and predict unexpected hypo-response or hyper-response, enabling personalized protocol optimization.

Tier 4 (environmental exposure adjustment) considers endocrine-disrupting chemicals, smoking, BMI, air pollution, and occupational exposures to correct predictive bias and improve safety through pre-treatment lifestyle interventions and OHSS prophylaxis.

**Figure 1:**
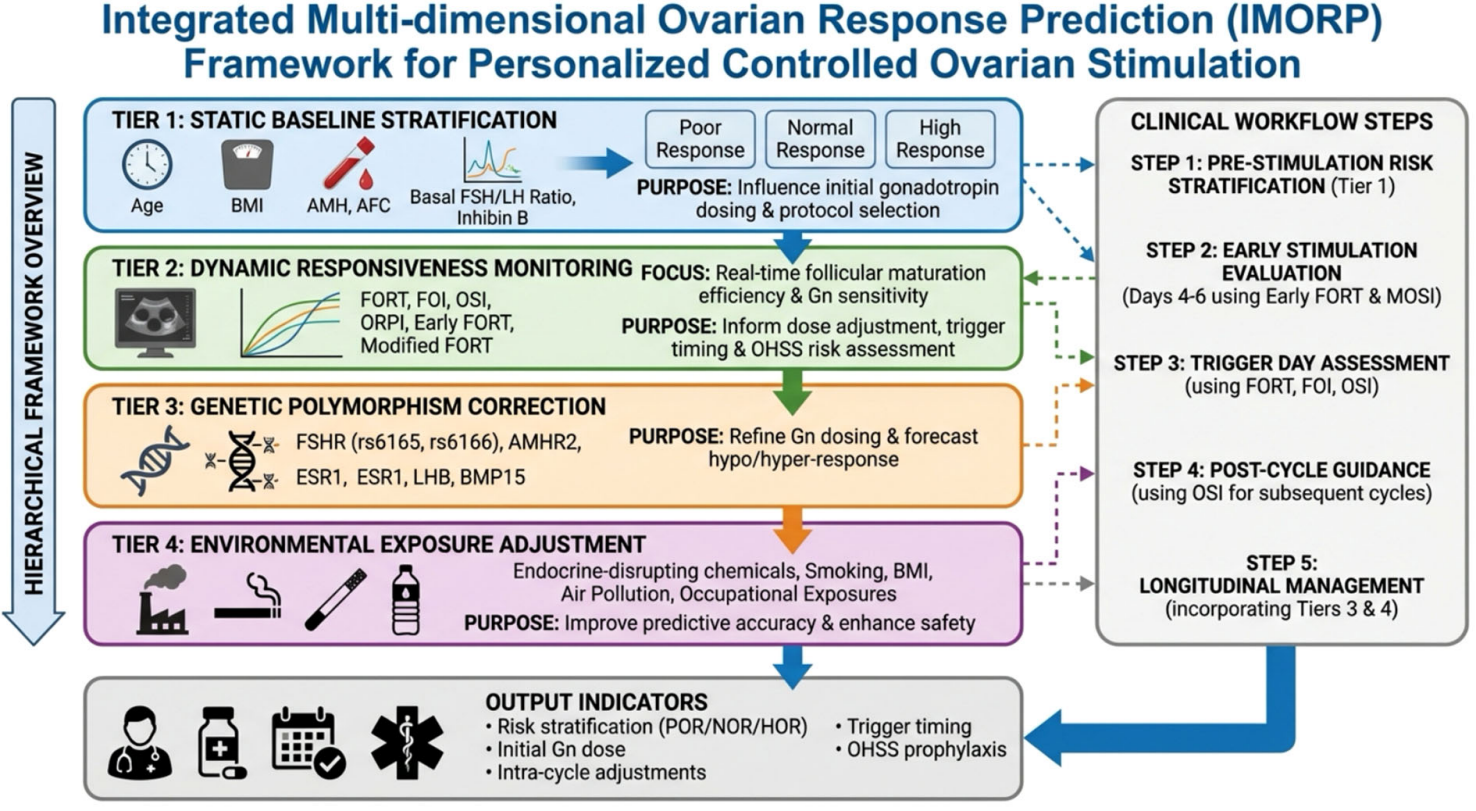
Integrated Multi-dimensional Ovarian Response Prediction (IMORP) framework for personalized COS. The framework comprises four hierarchical tiers (static markers, dynamic indices, genetic polymorphisms, and environmental factors) and a five-step clinical workflow (pre-stimulation, early stimulation, trigger day, post-cycle, and longitudinal management). Outputs include risk stratification, Gn dosing, cycle adjustments, trigger timing, and OHSS prevention.

The clinical workflow comprises five steps: (1) pre-stimulation risk stratification using Tier 1; (2) early stimulation (days 4–6) evaluation using early FORT and MOSI; (3) trigger day assessment using FORT, FOI, and OSI; (4) post-cycle guidance using OSI for subsequent cycles; and (5) longitudinal management integrating Tier 3 and Tier 4 information. This IMORP framework converts isolated indicators into a clinically actionable decision chain, moving ART from experience-based practice toward precision-guided individualized ovarian stimulation (**Figure 1**).

## 7 Clinical value and application strategies

The quality of the embryo critically determines pregnancy outcomes (106). Among euploid embryos, high-quality blastocysts with favorable morphology and normal morphokinetics exhibit superior developmental and implantation potential compared to their low-quality counterparts, leading to significantly higher clinical pregnancy rates and lower miscarriage risks. In contrast, aneuploid embryos, even when implantation occurs, rarely result in sustained pregnancy, yielding near-zero live birth rates (107).

Ovarian response primarily influences the total number of mature oocytes obtained, thereby indirectly affecting fertilization rates and the number of high-quality embryos available for transfer. However, studies have shown that among young women under 38 years of age, those with a low ovarian response (defined as an anti-Müllerian hormone level below the 10th percentile for their age) do not exhibit significant differences in blastocyst formation rate per oocyte, euploidy rate, or live birth rate per euploid embryo transfer compared with normal responders. In contrast, women with a high ovarian response (more than 15 oocytes retrieved) show a significantly lower oocyte maturation rate compared with normal responders (6–15 oocytes retrieved) (108). However, embryos derived from mature oocytes in the high-response group exhibit similar implantation potential as those with normal cleavage in the normal-response group. Therefore, a high ovarian response increases the absolute number of high-quality embryos available, providing more opportunities for achieving a successful pregnancy (109).

Even in the presence of high-quality embryos, a successful pregnancy is difficult to achieve without a synchronized endometrial microenvironment. Endometrial receptivity is a critical determinant of successful embryo implantation. Through the secretion of signaling molecules, the embryo engages in a molecular dialogue with the endometrium, which in turn expresses adhesion molecules, cytokines, and specialized structural features to accommodate the embryo, facilitating the orderly progression of apposition, adhesion, and invasion. Disruption of this endometrial–embryonic synchrony adversely affects the success rate of embryo transfer (110).

## 8 Conclusion

Ovarian response prediction is a cornerstone of successful ART. Static markers reliably evaluate ovarian reserve but cannot capture dynamic follicular responsiveness. Dynamic indices (FORT, FOI, OSI, ORPI) enable real-time assessment of stimulation efficiency, while genetic and environmental factors explain unexpected interindividual variability.

The IMORP framework proposed in this review unifies static, dynamic, genetic, and environmental dimensions into a clinically actionable workflow, addressing the lack of integrative models in current literature. By grading evidence and comparing predictive performance, we provide clinicians with clear guidance on indicator selection and application.

Future research should focus on large-scale prospective studies to validate dynamic index cutoffs, explore pharmacoepigenomics, and develop machine learning models that combine multi-omics and clinical data. Ultimately, this will drive the transition from experience-based to molecularly guided, individualized ovarian stimulation, maximizing live birth rates while minimizing complications such as OHSS.
